# Efficacy and safety of fruquintinib in the treatment of colorectal cancer: a systematic review and meta-analysis of studies in China

**DOI:** 10.3389/fphar.2025.1590782

**Published:** 2025-09-09

**Authors:** Yuan Feng, Yu Shu

**Affiliations:** ^**1**^ Department of Pharmacy, The Eighth Hospital of Wuhan, Wuhan, China; ^**2**^ Department of Oncology, The Eighth Hospital of Wuhan, Wuhan, China

**Keywords:** fruquintinib, colorectal cancer, overall survival, progression-free survival, safety, meta-analysis

## Abstract

**Objectives:**

To evaluate fruquintinib’s efficacy and safety in the treatment of colorectal cancer.

**Methods:**

Studies assessing fruquintinib for colorectal cancer were included. Outcomes were overall survival (OS) and progression-free survival (PFS), and adverse reactions. A random-effects model was employed, and sensitivity analysis assessed the stability of the results and potential heterogeneity. Review Manager 5.4 and STATA 15.0 were used for analysis.

**Results:**

Eleven studies with 2,367 patients were included. Fruquintinib significantly improved OS (HR: 0.69; 95% CI: 0.58, 0.81; P < 0.00001) and PFS (HR: 0.44; 95% CI: 0.30, 0.64; P < 0.0001). No significant increase in adverse events, serious adverse events, fatigue, or hypertension. However, sensitivity analysis suggested that the risk of hypertension might be unstable, requiring further validation.

**Conclusion:**

Fruquintinib improves OS and PFS in colorectal cancer patients without elevating the risk of overall or serious adverse events; however, its potential impact on hypertension risk requires further investigation. Due to limitations such as small sample size, missing data, and regional bias, larger, multicenter, double-blind RCTs are needed to validate these findings.

**Systematic review registration:**

https://www.crd.york.ac.uk/PROSPERO/, CRD420251002004.

## 1 Introduction

Colorectal cancer is one of the most common causes of illness and death worldwide. In 2020, the World Health Organization (WHO) reported more than 1.93 million new cases and 935,000 deaths, making it the third most common cancer and the second leading cause of cancer-related deaths (Sung et al., 2021). In Asia, colorectal cancer incidence has steadily increased, particularly in China, due to changes in lifestyle and diet, making it a major public health concern (Chen et al., 2016). Advances in early diagnosis and surgery have improved the prognosis of colorectal cancer patients, but treating metastatic colorectal cancer (mCRC) remains challenging. Standard treatment includes chemotherapy (e.g., FOLFOX, FOLFIRI) with targeted therapies (e.g., anti-EGFR, anti-VEGF monoclonal antibodies). However, drug resistance is a major issue, and later-line treatment options have limited efficacy (Van Cutsem et al., 2016). Developing new targeted therapies to improve survival in mCRC patients is a key focus of clinical research.

Fruquintinib is an oral, selective VEGFR-1/2/3 tyrosine kinase inhibitor developed by Chinese scientists. It interferes with the VEGF signaling pathway, suppressing tumor angiogenesis and exerting anti-tumor effects (Sun et al., 2014). Compared to multi-target inhibitors such as regorafenib, fruquintinib demonstrates superior VEGFR selectivity and reduced inhibition of kinases like PDGFR and FGFR, minimizing off-target toxicity (Wilhelm et al., 2011). Clinical research on fruquintinib began in 2014, with early trials showing its anti-tumor activity and tolerability in mCRC patients (Xu et al., 2017). The FRESCO study (NCT02314819), a randomized, double-blind, placebo-controlled phase III trial, confirmed fruquintinib’s efficacy in third-line mCRC treatment. The study demonstrated significant improvements in median overall survival (mOS: 9.3 vs 6.6 months, HR = 0.65) and progression-free survival (mPFS: 3.7 vs 1.8 months) (Li et al., 2018). Based on these results, fruquintinib was approved for third-line mCRC treatment in China in 2018 and by the FDA for global use in 2023 (Dasari et al., 2023).

Although fruquintinib has demonstrated significant clinical benefits in treating mCRC, existing studies have limitations, including small sample sizes and single-arm or single-center designs (Zhou et al., 2024; Deng et al., 2023; Zhang et al., 2022). The safety data for fruquintinib are inconsistent across studies and need further validation (Dai et al., 2022; Sun et al., 2021; Li et al., 2020). Yonatan et al. (2024) conducted a meta-analysis on the efficacy and safety of fruquintinib in refractory metastatic colorectal cancer, but it included only three studies, lacked significant real-world data, and provided low-quality evidence. This study seeks to consolidate existing data to assess fruquintinib’s efficacy and safety in treating colorectal cancer, thereby preliminarily integrating the currently available evidence evaluating the efficacy and safety of fruquintinib (either monotherapy or in combination) in Chinese patients with colorectal cancer (mainly metastatic/refractory) in real-world clinical practice, so as to depict the overall trends and potential problems in its use.

## 2 Methods

### 2.1 Literature search

This study adhered to the PRISMA 2020 guidelines (Page et al., 2021) and is registered with PROSPERO (CRD420251002004). A thorough search was performed in PubMed, Embase, Web of Science, and Cochrane databases until January 2025 for studies evaluating the effectiveness and safety of fruquintinib in the treatment of colorectal cancer. The following search terms were used: “fruquintinib” and “colorectal cancer”. Searching details as follows: ((“HMPL-013”[Supplementary Concept]) OR (fruquintinib)) AND ((“Colorectal Neoplasms” [Mesh]) OR ((((Colorectal Neoplasm) OR (Neoplasm, Colorectal)) OR (Colorectal Cancer)) OR (Colorectal Carcinomas))). We manually reviewed the reference lists. Articles were retrieved and assessed by two investigators, with any discrepancies resolved through consultation with the third author. A detailed literature search is presented in Supplementary Table S1.

### 2.2 Inclusion and exclusion criteria

Inclusion criteria:

P: colorectal cancer patients.

I: fruquintinib alone or in combination with other treatments.

C: treatments excluding fruquintinib.

O: overall survival (OS), progression-free survival (PFS), and adverse events (any adverse events refer to any adverse medical events of any grade related to the study drug, regardless of their severity; serious adverse events refer to adverse events of grade 3 or higher).

S: Randomized controlled trials (RCTs) and cohort studies

We excluded study protocols, unpublished studies, non-original research (e.g., meeting abstracts, corrections, replies), studies with inadequate data (survival or safety data unavailable directly or through transformation), and reviews.

### 2.3 Data abstraction

Two authors independently extracted the data, and with any discrepancies resolved through discussion between the two investigators: First author, year of publication, study duration, region, design, registration number, population, intervention, control, sample size, age, gender, follow-up period, OS, PFS, and safety outcomes were extracted. The corresponding authors were contacted to provide the missing details.

### 2.4 Quality evaluation

RCTs’ quality was assessed according to the Cochrane Handbook for Systematic Reviews of Interventions 5.1.0, with assessment across seven domains: randomization, allocation concealment, participant and personnel blinding, outcome assessment blinding, incomplete outcome data, selective reporting of outcomes, and other potential biases (Cumpston et al., 2019). Each domain was rated as low, high, or unclear risk. Studies with a greater number of “low risk” ratings were deemed of higher quality. The quality of cohort studies was assessed using the Newcastle-Ottawa Scale (NOS) (Wells, 2025), with high quality studies scoring 7–9 points (Kim et al., 2019). Two authors independently assessed, resolving any discrepancies through discussion.

### 2.5 Statistical analysis

Review Manager 5.4.1 was used for data synthesis. Hazard ratios (HR) with 95% confidence intervals (CI) were used to assess survival outcomes, and odds ratios (OR) with 95% CIs were computed for categorical variables. Heterogeneity across outcomes was evaluated using the chi-squared (χ^2^) test (Cochran’s Q) and the inconsistency index (Higgins and Thompson, 2002). Substantial heterogeneity was defined by a χ^2^ P value <0.1 or I^2^ > 50%. A random-effects model was used to calculate HR or OR. For outcomes with significant heterogeneity, a sensitivity analysis was conducted to evaluate the influence of individual studies on the overall HR or OR and identify potential sources of heterogeneity. Publication bias was assessed using Egger’s regression tests (Egger et al., 1997) in Stata 15.1 (Stata Corp, College Station, Texas, United States) for outcomes with more than 10 studies. P value <0.05 represents statistically significant publication bias.

## 3 Results

### 3.1 Literature retrieval, study characteristics, and baseline

**FIGURE 1:**
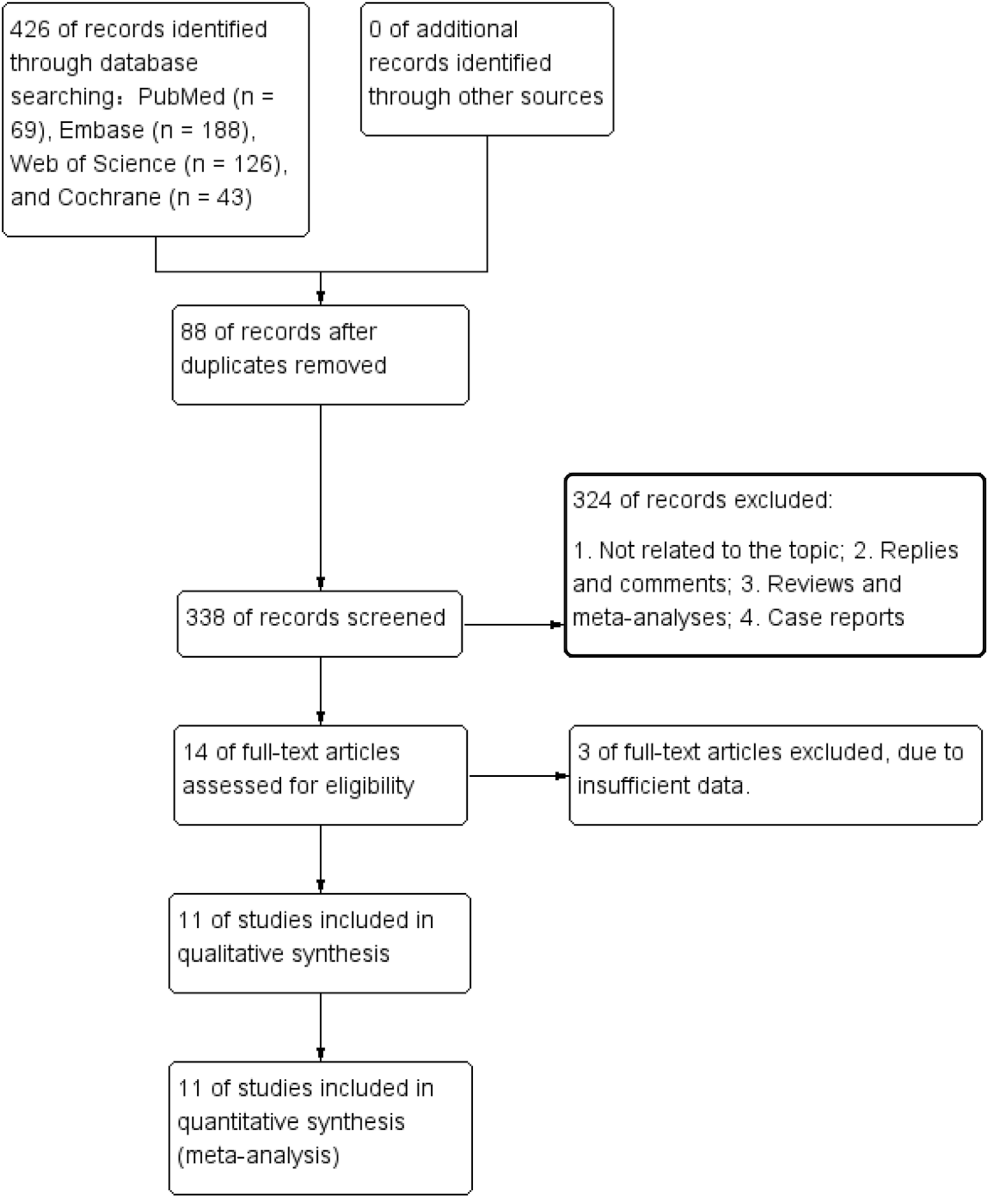
Flowchart of the systematic search and selection process.

**FIGURE 2:**
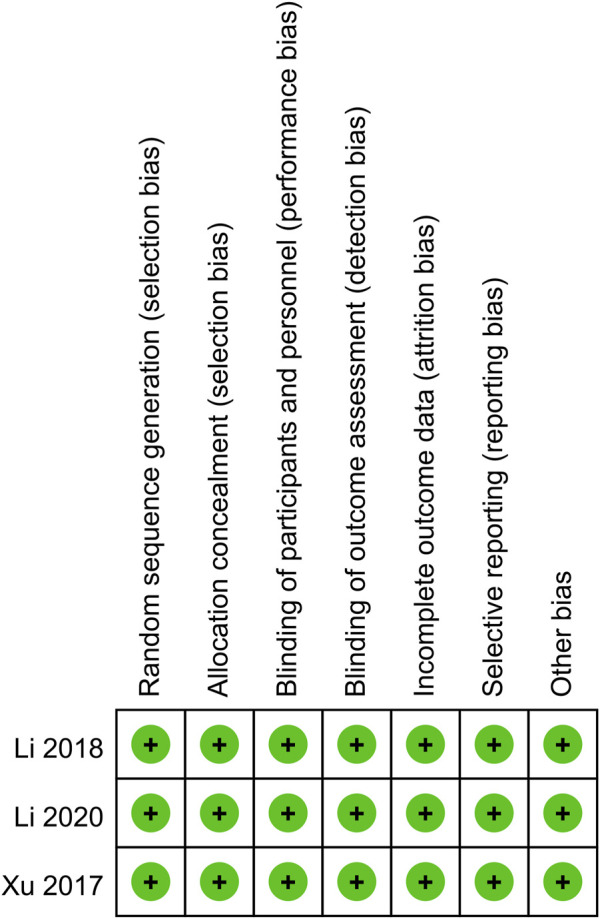
Details of the quality evaluation for included RCTs. All three RCTs had a low risk of bias (Green (+) represents low risk) in all listed domains.

The process of literature retrieval and selection was shown in Figure 1. A total of 426 studies were identified across PubMed (n = 69), Embase (n = 188), Web of Science (n = 126), and Cochrane (n = 43). After duplicates were removed, 338 titles and abstracts were screened. Ultimately, 11 studies (13 comparison groups) (Xu et al., 2017; Li et al., 2018; Zhou et al., 2024; Deng et al., 2023; Zhang et al., 2022; Dai et al., 2022; Sun et al., 2021; Li et al., 2020; Jin et al., 2022; Nie et al., 2022; Qin et al., 2021) involving 2,367 patients were included. Table 1 provides a summary of the characteristics, while Figure 2 presents the quality assessment. The included studies were all conducted in China between 2017 and 2024, including 3 RCTs and 8 observational cohort studies, reflecting a combination of experimental data and real-world evidence. The interventions showed significant heterogeneity: four studies (Li et al., 2018, Li et al., 2020; Xu et al., 2017; Zhang et al., 2022; with a total of 709 patients) evaluated fruquintinib monotherapy, mainly as third-line or later-line treatment for metastatic colorectal cancer (mCRC); the other seven studies evaluated combination therapy, including combination with PD-1 inhibitors (such as sintilimab, Sun et al., 2021 and Nie et al., 2022; with a total of 51 patients) in refractory microsatellite stable (MSS) mCRC, combination with chemotherapy regimens (such as raltitrexed/S-1, Zhou et al., 2024 and Zhou et al., 2024, with a total of 120 patients stratified by sex) in later treatment, and comparison with other targeted agents in mixed lines of treatment (such as regorafenib or bevacizumab, Deng et al., 2023; Jin et al., 2022; Dai et al., 2022; with a total of 308 patients). Patient populations were concentrated in advanced disease stages, with 10 studies enrolling only patients with metastatic colorectal cancer (stage IV). Key subgroup analyses included patients with liver metastases (Qin et al., 2021; n = 287) and those without liver metastases (Qin et al., 2021; n = 129). The majority of studies (9 of 11) included patients who had undergone multiple lines of therapy (≥2 prior regimens). Study sample sizes varied significantly, ranging from large RCTs (e.g., the FRESCO trial (Li 2018; n = 416) and its subgroup analyses (Qin et al., 2021; total n = 416), to moderate-sized cohorts (e.g., Zhang et al., 2022; n = 366, Jin 2022; n = 256), and smaller cohorts (e.g., Sun et al., 2021; n = 51, Nie et al., 2022; n = 72). This significant heterogeneity in interventions, patient populations, and study designs requires caution in interpreting the results of subsequent meta-analyses.

**TABLE 1:** Characteristics of included studies.

Study	Region	Study design	Population	Intervention	Control	Sample size		Follow-up	Mean/median age		Male		NOS score
						Fruquintinib	Control		Fruquintinib	Control	Fruquintinib	Control	
Dai et al., 2022	China	Cohort	Refractory metastatic colorectal cancer	Fruquintinib	Low-dose apatinib plus S-1	35	43	12.5months	53.00	52.00	20	30	8
Deng et al., 2023	China	Cohort	Metastatic colorectal cancer	Fruquintinib after the standard chemotherapy	Regorafenib after the standard chemotherapy	55	50	NA	61.00	63.00	42	38	9
Jin et al., 2022	China	Cohort	Metastatic colorectal cancer	Fruquintinib	Other TKIs	128	128	NA	56.09	56.15	78	68	8
Li et al., 2020	China	RCT	Previously treated metastatic colorectal cancer	Fruquintinib (5 mg once daily for 3 weeks, followed by 1 week off in 28-day cycles)	Placebo plus best supportive care	278	138	NA	55.00	57.00	158	97	—
Li et al., 2018	China	RCT	Previously treated metastatic colorectal cancer	Fruquintinib (5 mg once daily for 3 weeks, followed by 1 week off in 28-day cycles)	Placebo plus best supportive care	278	138	13.3 months	55.00	57.00	158	97	—
Nie et al., 2022	China	Cohort	Microsatellite stable metastatic colorectal cancer	Fruquintinib plus sintilimab as thirdline or above therapy	Regorafenib plus sintilimab as thirdline or above therapy	30	42	NA	56.00	59.00	13	23	9
Qin et al., 2021	China	Cohort	Patients with liver metastasis	Fruquintinib plus best supportive care	Placebo plus best supportive care	185	102	NA	NA	NA	109	74	7
Qin et al., 2021	China	Cohort	Patients without liver metastasis	Fruquintinib plus best supportive care	Placebo plus best supportive care	93	36	NA	NA	NA	49	23	7
Sung et al., 2021	China	Cohort	Refractory microsatellite stable metastatic colorectal cancer	Fruquintinib combined with PD-1 inhibitor	Regorafenib combined with PD-1 inhibitor	28	23	6.2 months	54.60	53.00	13	14	8
Xu et al., 2017	China	RCT	Patients with previously treated metastatic colorectal cancer	Fruquintinib plus best supportive care	Placebo plus best supportive care	47	24	NA	50.00	54.00	35	17	—
Zhang et al., 2022	China	Cohort	Metastatic colorectal cancer	Fruquintinib	Regorafenib	106	260	17.9 months	59.50	61.00	65	154	8
Zhou et al., 2024	China	Cohort	Patients with refractory metastatic colorectal cancer (male)	Raltitrexed, S-1 +fruquintinib	Raltitrexed, S-1 +bevacizumab	30	30	18.6 months	NA	NA	17	18	9
Zhao et al., 2024	China	Cohort	Patients with refractory metastatic colorectal cancer (female)	Raltitrexed, S-1 +fruquintinib	Raltitrexed, S-1 +bevacizumab	30	30	18.6 months	NA	NA	17	18	9

### 3.2 OS

**FIGURE 3:**
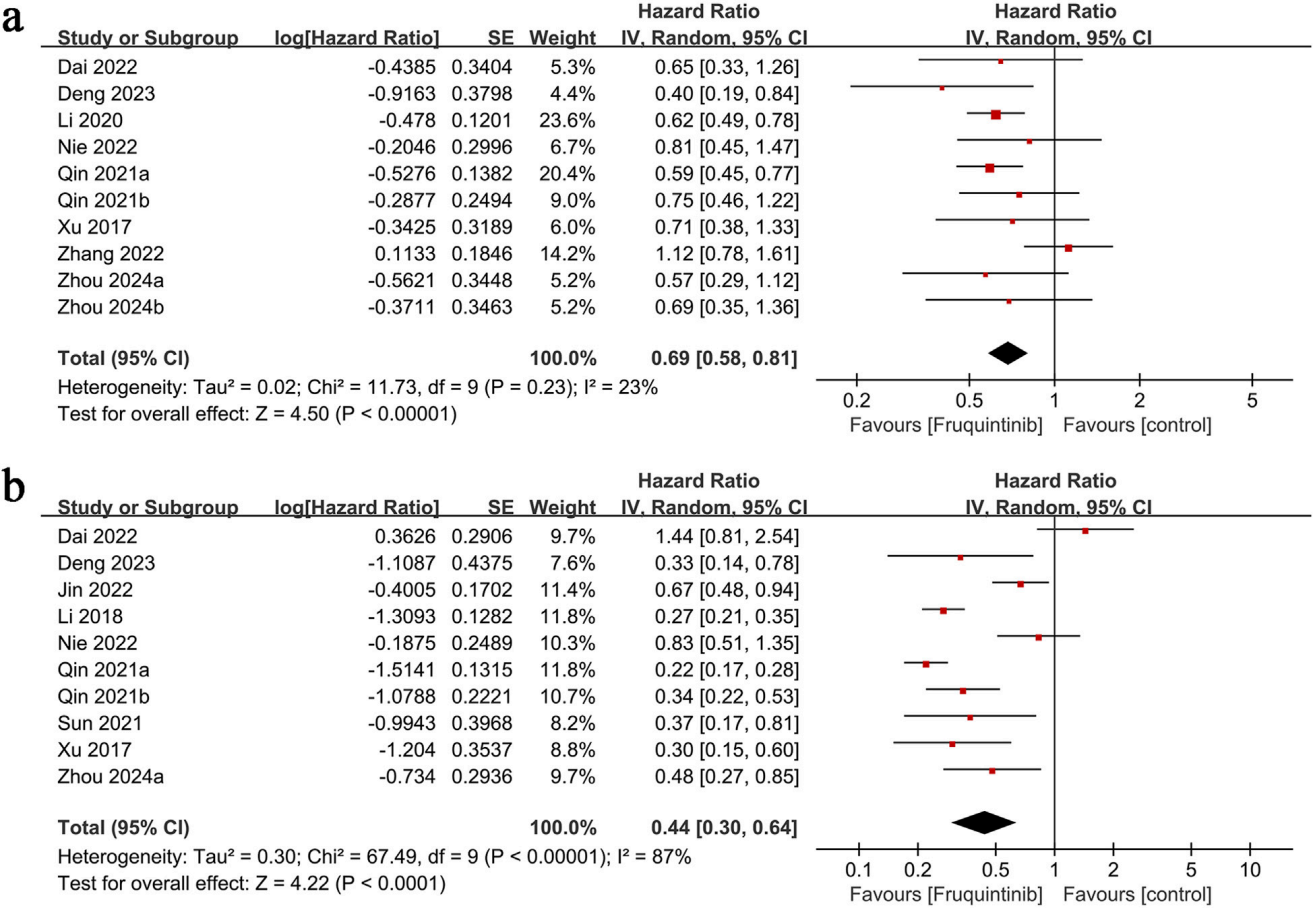
Forest plots of **(a)** OS, **(b)** PFS.

OS results were derived from 10 comparison groups. The meta-analysis demonstrated significantly longer OS in the fruquintinib group (HR: 0.69; 95% CI: 0.58, 0.81; P < 0.00001), with no significant heterogeneity (I^2^ = 23%, P = 0.23) (Figure 3a).

### 3.3 PFS

PFS data from 10 comparison groups demonstrated significantly prolonged PFS in the fruquintinib group (HR: 0.44; 95% CI: 0.30, 0.64; P < 0.0001) with notable heterogeneity (I^2^ = 87%, P < 0.00001) (Figure 3b).

### 3.4 Any adverse events

**FIGURE 4:**
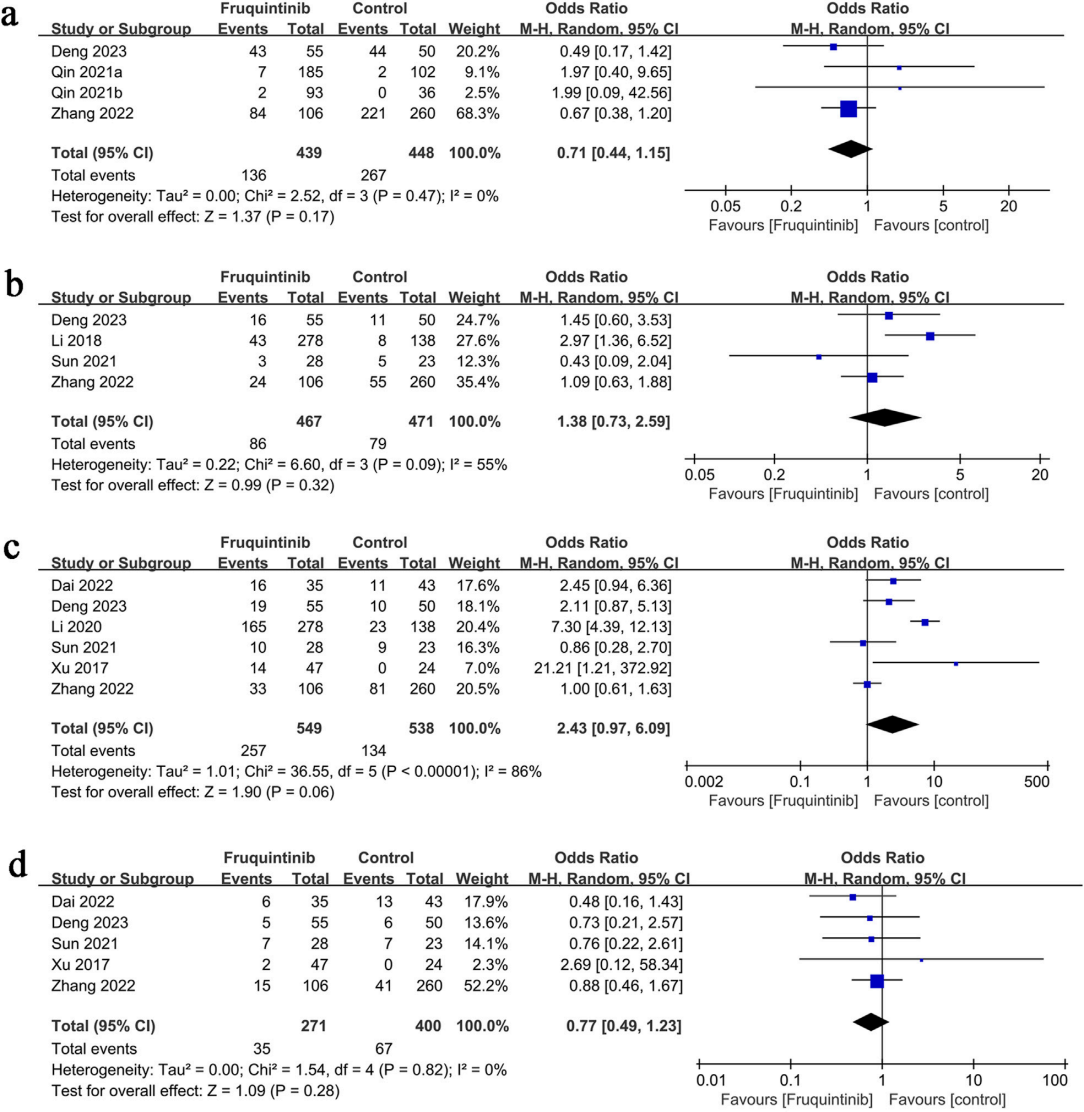
Forest plots of **(a)** any adverse events, **(b)** serious adverse events, **(c)** hypertension, and **(d)** fatigue.

The analysis of adverse events included four groups, revealing no significant difference in risk between them (OR: 0.71; 95% CI: 0.44, 1.15; P = 0.17) and no heterogeneity (I^2^ = 0%, P = 0.47) (Figure 4a).

### 3.5 Serious adverse events

The analysis of serious adverse events included four groups, revealing no significant difference in risk between them (OR: 1.38; 95% CI: 0.73, 2.59; P = 0.32), with notable heterogeneity (I^2^ = 55%, P = 0.09) (Figure 4b).

### 3.6 Hypertension

Hypertension results involved six groups, showing no significant difference in risk between them (OR: 2.43; 95% CI: 0.97, 6.09; P = 0.06), with notable heterogeneity (I^2^ = 86%, P < 0.00001) (Figure 4c).

### 3.7 Fatigue

Fatigue results comprised five groups, with no significant difference in risk (OR: 0.77; 95% CI: 0.49, 1.23; P = 0.28) and no heterogeneity (I^2^ = 0%, P = 0.82) (Figure 4d).

### 3.8 Sensitivity analysis and publication bias

**FIGURE 5:**
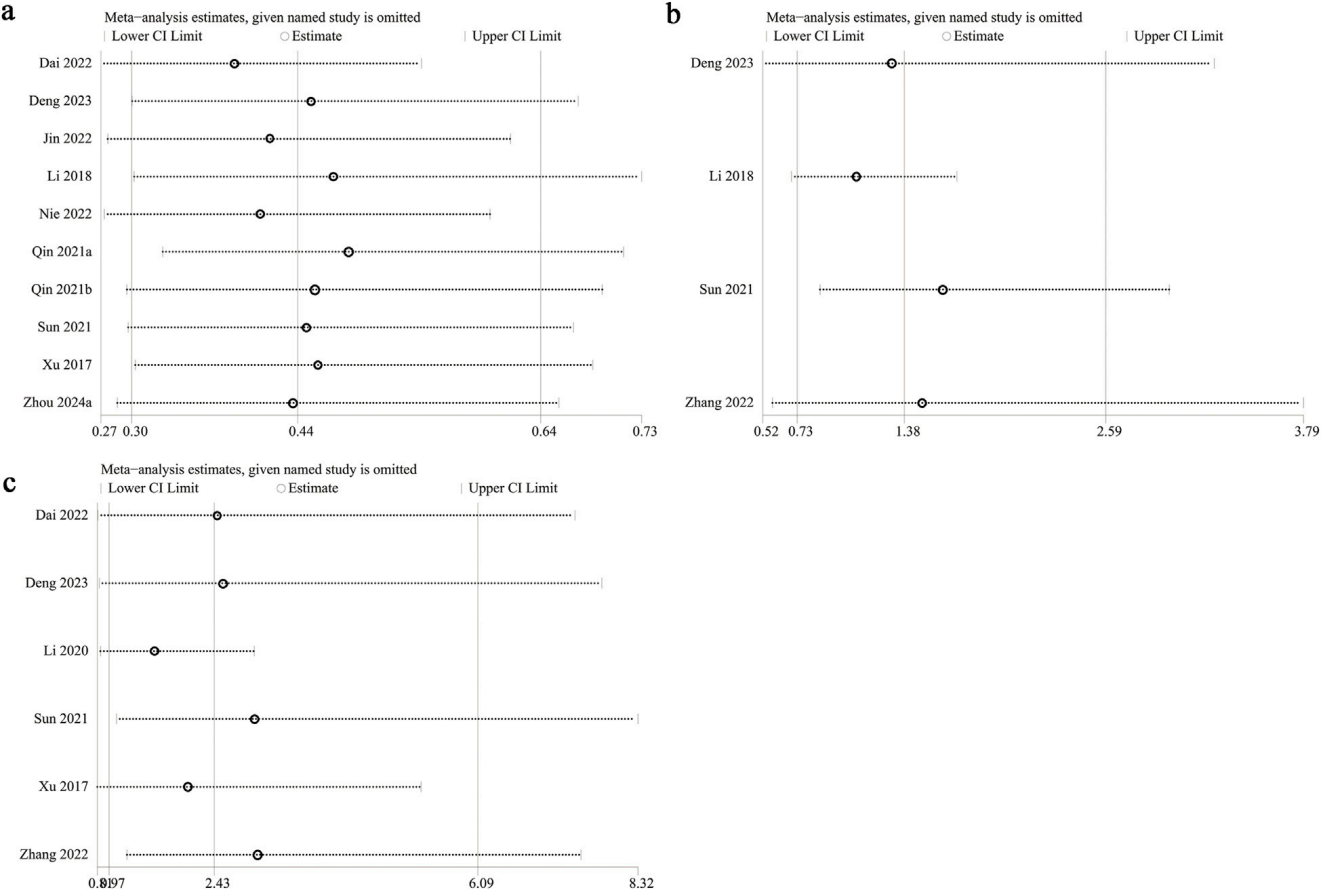
Sensitivity analysis of **(a** PFS, **(b)** serious adverse events, and **(c)** hypertension.

Sensitivity analysis was performed on PFS, serious adverse events, and hypertension to evaluate the impact of individual studies on the overall HR or OR by sequentially excluding each study. The analysis demonstrated consistent estimates for PFS (Figure 5a) and serious adverse events (Figure 5b) following the exclusion of each study. However, excluding data from Sun et al. (2021) (OR: 2.99; 95% CI: 1.07, 8.32) and Zhang et al. (2022) (OR: 3.03; 95% CI: 1.22, 7.52) shifted hypertension risk from nonsignificant to significant (Figure 5c), indicating that fruquintinib may elevate the risk of hypertension. Excluding Li 2018 (Li et al., 2018) reduced heterogeneity of serious adverse events from 55% to 0%, identifying it as a key source of variability. Similarly, excluding Li 2020 (Li et al., 2020) decreased hypertension heterogeneity from 86% to 52%, highlighting it as another major source of variability. Primary data of sensitivity analysis of hypertension was presented in Supplementary Figure S1.

**FIGURE 6:**
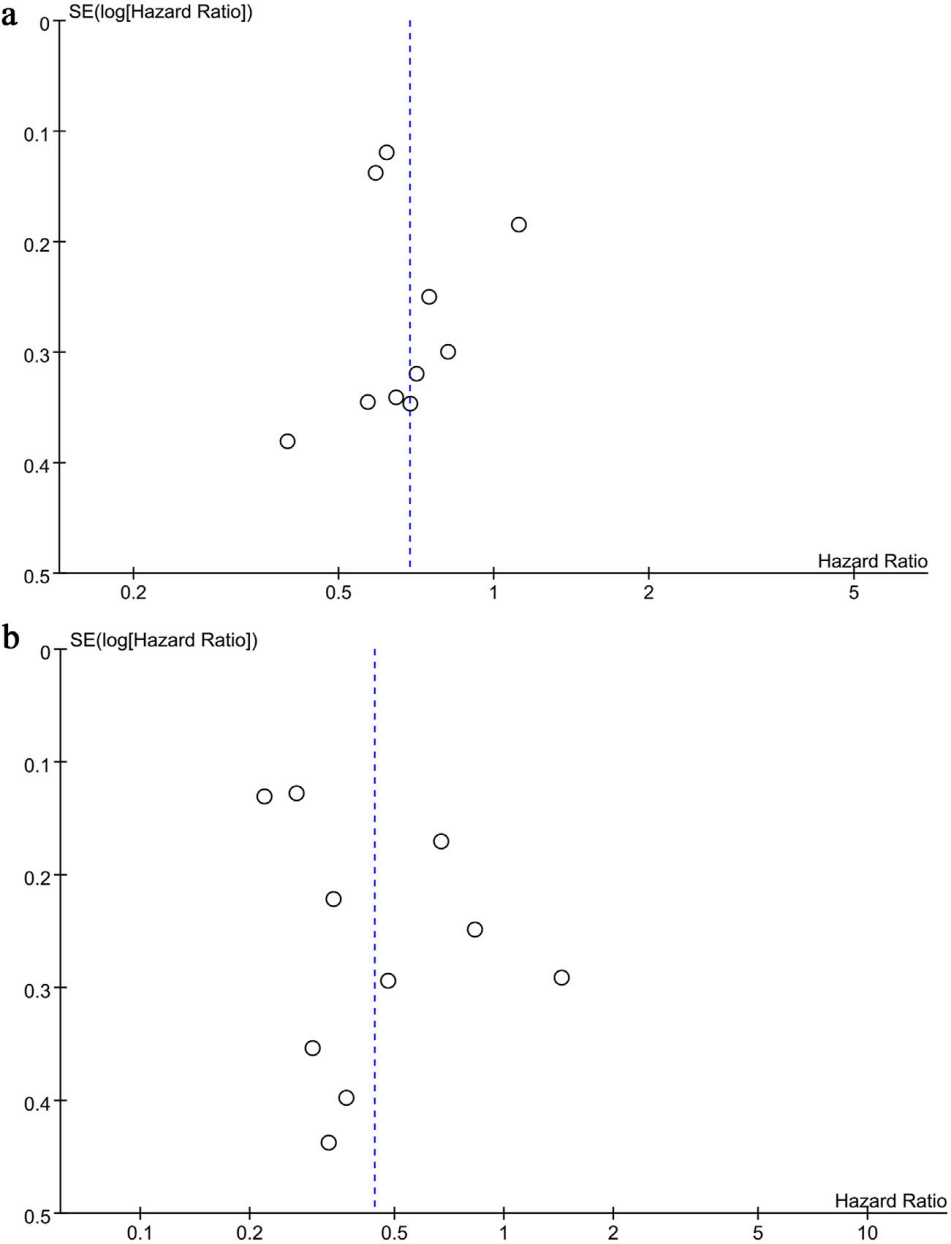
Funnel plots of **(a)** OS, **(b)** PFS.

Funnel plots and the Egger test were used to evaluate publication bias of OS and PFS. Both plots were symmetrical (OS: Figure 6a; PFS; Figure 6b), indicating no significant bias, as confirmed by the Egger test (OS: P = 0.927, PFS: P = 0.179).

## 4 Discussion

Colorectal cancer is the third most prevalent malignancy worldwide. The prognosis for mCRC remains poor, as resistance to chemotherapy and targeted therapies limits survival (Ciracì et al., 2025). Anti-angiogenic therapy is a critical strategy, with vascular endothelial growth factor receptor (VEGFR) inhibitors playing a pivotal role in blocking tumor angiogenesis (Zhao et al., 2024). Fruquintinib, a selective VEGFR-1/2/3 tyrosine kinase inhibitor developed in China, became a key option for later-line treatment following its approval for third-line mCRC therapy in 2018 (Li et al., 2018). Several pivotal studies have solidified fruquintinib’s clinical role. The FRESCO study (NCT02314819) demonstrated that fruquintinib significantly extended median overall survival (mOS: 9.3 vs 6.6 months, HR = 0.65) and progression-free survival (mPFS: 3.7 vs 1.8 months) compared to placebo (Qin et al., 2021). The global multicenter FRESCO-2 study further validated its efficacy (mOS: 7.4 vs 4.8 months, HR = 0.66), with consistent benefits observed across subgroups, including those previously treated with anti-VEGF/EGFR therapies. These results contributed to its FDA approval in 2023 (Dasari et al., 2023). Furthermore, combining fruquintinib with immunotherapy (e.g., sintilimab) exhibited synergistic potential in microsatellite stable (MSS) mCRC, offering new approaches for “cold tumors” (Wang et al., 2024).

Fruquintinib is currently being investigated for frontline treatment. Compared to antivascular agents like bevacizumab, its selective VEGFR inhibition and short half-life may minimize off-target toxicity. However, adverse events such as hypertension remain a concern (Xu et al., 2017). While fruquintinib demonstrates both efficacy and potential in colorectal cancer treatment, its optimal use and safety profile require further evaluation (Syaj and Saeed, 2024; Stucchi et al., 2024). This study aims to consolidate existing clinical data through meta-analysis to provide robust evidence on fruquintinib’s efficacy and safety in colorectal cancer treatment.

This meta-analysis of 11 studies confirmed that fruquintinib significantly improved OS and PFS in colorectal cancer patients without increasing the incidence of overall or serious adverse events. These results are consistent with the FRESCO series. Our findings align with those of Yonatan et al. (2024), who demonstrated that fruquintinib enhances survival and tumor response in patients with refractory metastatic colorectal cancer. In contrast to our study, Yonatan et al. reported a higher incidence of grade 3 or greater adverse events. However, their meta-analysis included only three RCTs, providing limited evidence for safety conclusions. Our study, integrating data from six real-world cohort studies in addition to RCTs, significantly expands the sample size and enhances the extrapolation of conclusions to broader clinical practice settings. Our sensitivity analysis indicated instability in hypertension risk, suggesting that fruquintinib may increase the risk of hypertension, though further real-world data are necessary to confirm this. Based on the observed OR of 2.43 and the fluctuation observed in sensitivity analysis, we recommend assessing baseline blood pressure and cardiovascular history before initiating fruquintinib. High-risk patients, such as those with chronic kidney disease, should be closely monitored for blood pressure fluctuations. Even for patients with poor prognosis, weekly monitoring is advised.

This meta-analysis suggests that fruquintinib may elevate hypertension risk, consistent with the 21.6% incidence of grade 3 hypertension in the FRESCO study. The likely mechanism involves increased vascular resistance and reduced nitric oxide synthesis due to VEGFR inhibition (Patell et al., 2024). We recommend assessing baseline blood pressure and cardiovascular history before prescribing fruquintinib. High-risk patients, such as those with chronic kidney disease, should be closely monitored for blood pressure fluctuations. Even for patients with poor prognosis, weekly monitoring is advised, with antihypertensive treatment initiated if readings reach ≥140/90 mmHg (Li et al., 2024). According to the FRESCO-2 protocol, grade 3 hypertension should be managed by suspending treatment until it improves to grade 1, followed by dose reduction to 4 mg or 3 mg (Dasari et al., 2023). Combining fruquintinib with NSAIDs or other pressor drugs should be avoided. Patients should follow a low-salt diet, engage in exercise, and biomarkers (e.g., VEGF gene polymorphisms) should be investigated to predict hypertension risk and guide personalized dosing strategies (Ayala-de Miguel et al., 2024; Ding et al., 2020).

Fruquintinib is a selective inhibitor of VEGFR-1/2/3 tyrosine kinases that disrupts the VEGF signaling pathway, hindering tumor angiogenesis and exerting anti-tumor effects. The VEGF pathway plays a pivotal role in tumor angiogenesis. Fruquintinib competitively binds to the ATP-binding site of VEGFR-1/2/3, inhibiting autophosphorylation and downstream signaling (e.g., PI3K/AKT and MAPK), thereby suppressing endothelial cell proliferation, migration, and angiogenesis (Hicklin and Ellis, 2005; Ferrara et al., 2003). Preclinical studies show that fruquintinib’s IC50 for VEGFR-2 is 1.6 nM, lower than regorafenib’s (IC50 = 4.2 nM), indicating a more potent VEGFR inhibition (Sun et al., 2014). Compared to multi-target inhibitors like regorafenib, fruquintinib exhibits higher selectivity for VEGFR-1/2/3, but weaker effects on other kinases, such as PDGFR and FGFR. For instance, the IC50 for PDGFR-β is 100 nM, and for FGFR-1, it exceeds 1,000 nM, much higher than for VEGFR (Wilhelm et al., 2011). This selectivity may minimize off-target toxicity (e.g., hand-foot syndrome, bone marrow suppression), enhancing clinical tolerability (Li et al., 2018). Combined with the lower risk of fatigue observed in this meta-analysis, these findings support the theoretical advantage of fruquintinib’s high selectivity in potentially reducing off-target toxicity compared to multi-targeted inhibitors like regorafenib.

In a colorectal cancer xenograft model, fruquintinib reduced tumor growth by 68%. When combined with PD-1 inhibitors, it enhanced CD8^+^ T cell infiltration and reversed immunosuppression, suggesting potential synergy with immunotherapy (Yang et al., 2023). Bevacizumab, which targets only VEGF-A, is prone to resistance due to bypass activation (e.g., VEGF-C/D). In contrast, fruquintinib may overcome this limitation by inhibiting VEGFR-1/2/3 (Ellis and Hicklin, 2008). Moreover, regorafenib’s inhibition of multiple kinases (e.g., RAF, KIT) may lead to greater off-target toxicity, such as hand-foot syndrome. In comparison, fruquintinib’s high selectivity improves its safety profile (Grothey et al., 2013).

This meta-analysis has several limitations. First, the interventions and control groups across the included studies were not uniform (including fruquintinib monotherapy and various combination therapies), potentially contributing to heterogeneity. Additionally, variations in the definitions of PFS may further explain this heterogeneity. Population differences, especially in tumor staging, may also influence the therapeutic effects of fruquintinib. Due to the limited number of studies, this analysis could not evaluate surgery-related outcomes or chemotherapy response. Furthermore, subgroup analyses based on factors such as chemotherapy regimens, cycle number, age, and tumor pathology were not feasible due to insufficient data. Besides, the absence of age data in some included studies limited our ability to perform age-specific subgroup analyses and comprehensively evaluate the influence of age on outcomes, particularly safety profiles. Finally, since all included studies were from China, the lack of data from other populations limits the generalizability of our findings. Despite these limitations, this meta-analysis offers a thorough review of existing clinical studies, confirming the efficacy and safety of fruquintinib in treating colorectal cancer. However, larger-scale, multicenter, double-blind randomized controlled trials are needed for further validation.

## 5 Conclusion

Fruquintinib improves OS and PFS in colorectal cancer patients without elevating the risk of overall or serious adverse events; however, its potential impact on hypertension risk requires further investigation. Due to limitations such as small sample size, missing data, and regional selection bias, larger, multicenter, double-blind RCTs are required to validate these findings.

## Data Availability

The original contributions presented in the study are included in the article/Supplementary Material, further inquiries can be directed to the corresponding author.
