# Supplementary material for: Efficacy and safety of fruquintinib in the treatment of colorectal cancer: a systematic review and meta-analysis of studies in China
Source: Front Pharmacol. 2025 Sep 9;16:1590782. doi: 10.3389/fphar.2025.1590782 (PMC12454978; doi:10.3389/fphar.2025.1590782)

Table S1. Literature Search Strategy.

Pubmed-69

(("HMPL-013" [Supplementary Concept]) OR (fruquintinib)) AND (("Colorectal Neoplasms"[Mesh]) OR ((((Colorectal Neoplasm) OR (Neoplasm, Colorectal)) OR (Colorectal Cancer)) OR (Colorectal Carcinomas)))

Embase-188


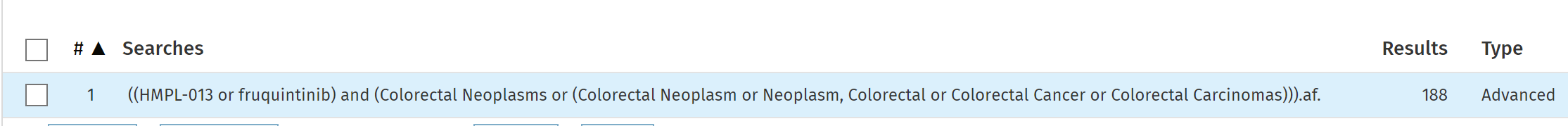


Cochrane-43


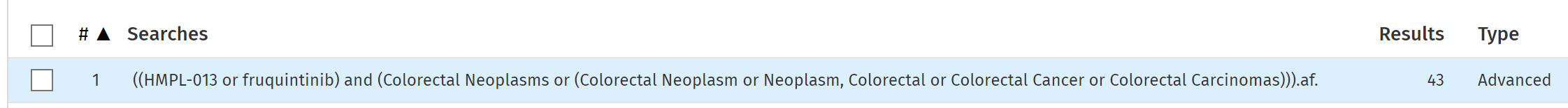


WOS-126

((HMPL-013) OR (fruquintinib)) AND ((Colorectal Neoplasms) OR ((((Colorectal Neoplasm) OR (Neoplasm, Colorectal)) OR (Colorectal Cancer)) OR (Colorectal Carcinomas))) (Topic) and Preprint Citation Index (Exclude – Database)

Figure S1. Primary data of sensitivity analysis of hypertension.


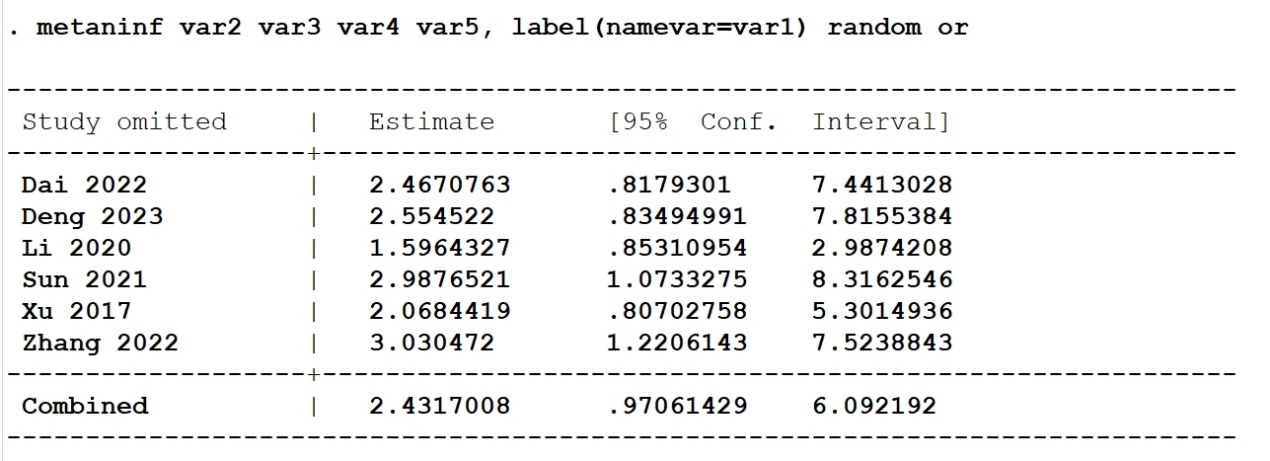

Supplement: Supplementary file 1 [file DataSheet1.docx]
